# Infertility management according to the Endometriosis Fertility Index in patients operated for endometriosis: What is the optimal time frame?

**DOI:** 10.1371/journal.pone.0251372

**Published:** 2021-05-12

**Authors:** Alexandre Bailleul, Julien Niro, Joseph Du Cheyron, Pierre Panel, Arnaud Fauconnier

**Affiliations:** 1 Research Unit EA7285, Risk and Safety in Clinical Medicine for Women and Perinatal Health, Versailles St-Quentin University, Montigny-le-Bretonneux, Versailles, France; 2 Department of Gynecology & Obstetrics, Centre Hospitalier André Mignot, Versailles, France; 3 Clinical Research Department, Centre Hospitalier Intercommunal de Poissy-Saint-Germain-en-Laye, Poissy, France; 4 Department of Gynecology & Obstetrics, Centre Hospitalier Intercommunal de Poissy—Saint-Germain, Poissy, France; University of Insubria, ITALY

## Abstract

**Introduction:**

The Endometriosis Fertility Index (EFI) is a validated score for predicting the postoperative spontaneous pregnancy rate in patients undergoing endometriosis surgery. However, the practical use of the EFI to advise patients about postoperative fertility management is unclear.

**Materials and methods:**

All patients participating in the ENDOQUAL study–a prospective observational bi-center cohort study conducted between 01/2012 and 06/2018–who underwent surgery for infertility were asked to complete a questionnaire collecting time and mode of conception. Statistical analysis was performed with the Fine and Gray model of competing risks and analysis of fertility according to the EFI.

**Results:**

Of the 234 patients analyzed, 104 (44.4%) conceived postoperatively including 58 (55.8%) spontaneous pregnancies. An EFI of 0–4 for spontaneous pregnancies was associated with a lower cumulative pregnancy incidence compared to an EFI of 5–10 (52 versus 34 pregnancies respectively, Subdistribution Hazard Ratio (SHR) = 0.47; 95% CI [0.2; 1.1]; p = 0.08). An EFI of 0–4 was associated with a higher cumulative pregnancy rate for pregnancies obtained by artificial reproduction technology (ART), compared to an EFI of 5–10 (12 versus 6 pregnancies respectively, SHR = 1.9; CI95% [0.96; 3.8]; p = 0.06). Fecundability decreased from 12 months for EFI 0–4 and from 24 months for EFI 5–10.

**Conclusion:**

Our analysis suggests that patients with an unfavorable EFI (≤4) have more ART pregnancies than patients with a favorable EFI (≥5) and should be referred for ART shortly after surgery. Patients with a favorable EFI may attempt spontaneous pregnancy for 24 months before referral.

## Introduction

Endometriosis is a benign gynecological disease which affects 6 to 10% of women of reproductive age [1]. This extrinsic localization of endometrial tissue is responsible for chronic inflammation generating in anatomical pelvic modifications [2]. However, the etiopathogenesis of endometriosis is a multifactorial process resulting in a heterogeneous disease [3]. Patients usually present chronic pelvic pain, infertility or impaired quality of life; these symptoms can be associated with one another [4]. Spontaneous pregnancy rates in women with endometriois-related infertility have been reported at around 10% [5]. While medical treatments can be effective in the management of endometriosis, surgery has been demonstrated to be a valid therapeutic tool [6]. Surgical resection of the lesions can double the chances of woman natural conception [7, 8].

The Endometriosis Fertility Index (EFI) was developed to predict the spontaneous pregnancy rate in women 3 years after surgery for endometriosis [9]. This multifactorial score includes criteria based on the patient’s characteristics (age, duration of infertility, pregnancy history), intra-operative lesion description (American Society for Reproductive Medicine (ASRM), American Fertility Society (AFS) Endometriosis Score) and a functional post-operative score (Least Function (LF) Score). The EFI is the sum of the surgical and historical factors, and ranges from 0 to 10. The rate of spontaneous pregnancies is greater in women with higher EFI scores; cumulative non-ART pregnancy at 36 months was found to be 10% (95%CI: 3, 16; P< 0.001) for women with an EFI of 0–2, and 69% (95%CI: 58, 79; P< 0.001) for women with an EFI of 9–10 [10]. The EFI was described by the World Endometriosis Society (WES) in 2017 as being a robust and clinically valid score to predict fertility after surgery in patients with endometriosis [11]. Consequently, the French College of Gynecologists and Obstetricians (CNGOF) recommends that the EFI be used for guiding the post-operative strategy. However, the EFI is a predictive tool rather than a therapeutic decision-making tool and clinical interpretation to determine the best approach for post-operative fertility is ambiguous [12]. In the literature, the decisional threshold indicating a good likelihood of spontaneous pregnancy is generally taken to be 5 [13–15]. However, the current interest is to determine the role of the EFI in the post-operative management of patients with endometriois-related infertility: i.e., spontaneous conception or assisted reproductive technology (ART) and the optimal time frame for each.

The objective of the present study was therefore to assess the value of the EFI in deciding the optimal time to refer patients for ART after surgery for endometriosis.

## Materials and methods

### Data source

The patients analyzed in the current study were all part of the ENDOQUAL cohort. ENDOQUAL was an observational, prospective, bi-center study (CHI Poissy—St Germain en Laye (CHIPS) and CH Mignot de Versailles (CHV)) [16]. The aim of ENDOQUAL was to investigate the impact of different therapeutic modalities (medical treatment, surgical treatment, ART) on quality of life and fertility by collecting clinical information on volunteer endometriosis patients. ENDOQUAL began on January 01, 2012 and the EFI was routinely measured until January 01, 2017. The study was approved by the Southeast IV Ethics Committee (Sud-Est, n°18/002) in France and the French National Committee for Information Technology and Individual Liberties (N°906 253). Our study was purely observational and involved no intervention. As such, no written informed consent was required under French law (the Huriet-Serusclat Act of December 20, 1998). Nevertheless, all the patients received information about the study and were free to participate or not.

### Study design

All patients with endometriosis-related infertility (with or without pain component) and women with endometriosis with an immediate desire to conceive who underwent surgery between 01/01/2012 and 31/06/2018 were included. In our expert centers and in accordance with French gynecologist recommendations, women who require immediate in vitro fertilization (IVF) (e.g., for major male factor or tubal anomalies) are not eligible for surgery.

Non-inclusion criteria were women older than 45 years, radical surgery (hysterectomy, bilateral adnexectomy), included in the medical arm of the ENDOQUAL study (hormonal treatment or ART), or operated on for an indication other than infertility and without an immediate desire to conceive.

The objective of the surgical procedure was therefore to improve spontaneous fertility. All the women underwent a laparoscopic fertility-sparing resection of endometriosis to remove all endometriosis implants and adhesions. Following the surgery, the women attempted to conceive spontaneously. No specific recommendations were given during the study period about whether to refer a patient for ART according to their EFI score.

Patients were excluded if the histology was negative for endometriosis, if they had a history of pelvic surgery known to be at high risk of adhesion (laparotomy), or if they had a history of multiple (> 2) pelvic surgical procedures for endometriosis.

The primary endpoint was the time of occurrence of a pregnancy (whatever its outcome) according to the mode of conception: spontaneous or by ART (stimulation, insemination, or IVF). The secondary endpoint was the postoperative fecundability, which is defined as the probability of achieving a pregnancy within one menstrual cycle.

### Follow-up

Fertility outcomes were collected with two questionnaires survey: (i) one sent one year after the surgery according to the cohort study protocol, (ii) and a second one sent in January 2020. If no reply was received, contact was made by telephone after checking for any change of address. When a patient was lost to follow-up, her doctor was contacted. The questionnaires covered the following information: how long the patient had attempted to achieve pregnancy, any attempts at ART, and, for each pregnancy obtained after surgery, the date and means of conception (spontaneous, ART) and its outcome (miscarriage, therapeutic abortion, voluntary abortion, birth).

### EFI score calculation

As the EFI was not used in regular practice between 2012 and 2017, the EFI score was calculated *a posteriori* for women who underwent surgery before 2017 (ENDOQUAL-1) by collecting the relevant data from the detailed operative report of the database and the textual operative reports. After 2017 (ENDOQUAL-2), the EFI was included in the Case Report Form (CRF) of the ENDOQUAL study. To avoid calculation bias and confirm the reproducibility of the EFI, we compared the *a posteriori* calculation of the EFI scores with the EFI scores from women included after 2017 [17].

The maximum EFI score is ten: five points are based on the patient’s characteristics (such as age, duration of infertility, and history of pregnancy); two points on rASRM staging; and the remaining three points on qualitative assessment by the surgeon (adnexal LF score) on completion of the procedure [9]. For example, a 28-year-old nulliparous patient, who has been infertile for 1 year, with only superficial endometriosis without adnexal lesions, has an EFI score of 9. On the contrary, a 38-year-old nulliparous patient who has been infertile for more than 3 years, with deep endometriosis (such as a complete posterior cul-de-sac obliteration and bilateral endometrioma), has an EFI score of 3 after the removal of all implants and adhesion.

### Statistical analysis

The postoperative pregnancy rate and time to pregnancy were analyzed according to the conception mode (spontaneous or ART) for two groups of patients: patients with an EFI of 0–4 and those with an EFI of 5–10. The time from the beginning of the observation period was the date of the surgery.

Patient characteristics were recorded and compared according to time to pregnancy, age, endometriosis classification (ASRM, AFS Endometriosis Score, LF Score), EFI, tobacco use, Body Mass Index (BMI), and Anti-Müllerian Hormone (AMH) levels.

As patients may have two competing events (spontaneous conception and ART conception), we used a competing risk approach to explore the relation between EFI and fertility [18]. Modeling cumulative incidence curves by the semi-parametric Fine and Gray model (1999) allowed the calculation of the risk function associated with the cumulative incidence function (Subdistribution Hazard Ratio—SHR) by a competing risks multivariate analysis. [19, 20]. This analysis compared the cumulative pregnancy curves with various co-variables. This model (Fine and Gray / SHR) was the most appropriate to analyzed and interpreted our data and responded to our primary endpoint.

Cause-specific hazard ratios (CSHR) is using a Cox proportional hazard model, in which patients who experience other events are treated as censored for the event of interest. Sub-distribution hazard ratios (SHR) is using the Fine and Gray model, in which woman who experience other events are treated as immune (i.e., “cured” and remaining in the risk sets) to the event of interest.

Finally, the objective was to determine the time lost before conception between the two groups. Analyses were based on Area Under the Curve (AUC) and fecundability [21–23]. For both these analyses, we focused only on spontaneous conception and patients were censored when they were lost to follow-up or became pregnant with ART.

Calculating the AUC in censored data provides the Restricted Mean Survival Time (RMST). This time is equivalent to the area under the Kaplan-Meier curve from the start of the study to an interest time point (36 months in our study, i.e., the maximum follow-up of the patients’ fertility after surgery in accordance with Adamson et al. [9]). To quantify the difference between the two EFI groups, the analysis was performed on the difference in RMSTs. It is interpreted as a gain or loss of event-free survival time for a given period. The RMST, in our analysis, corresponds to a Restricted Mean Conception Time (RMCT). A decomposition of 6-month postoperative intervals aimed to find the time from which patients with an EFI of 0–4 had a loss of chance for spontaneous conception compared to the other group.

Fecundability is the probability of conception per cycle during an interval [24]. The estimate of the monthly fecundability $\hat{f}$ was obtained by dividing the number of conceptions observed *Ci* by the sum of the person-months of exposure *Ci* during an interval:

$$\hat{f}=\frac{\Sigma Ci}{\Sigma Ti}$$

All statistical analysis was performed using available software (R—1.2.5019).

## Results

**Fig 1:**
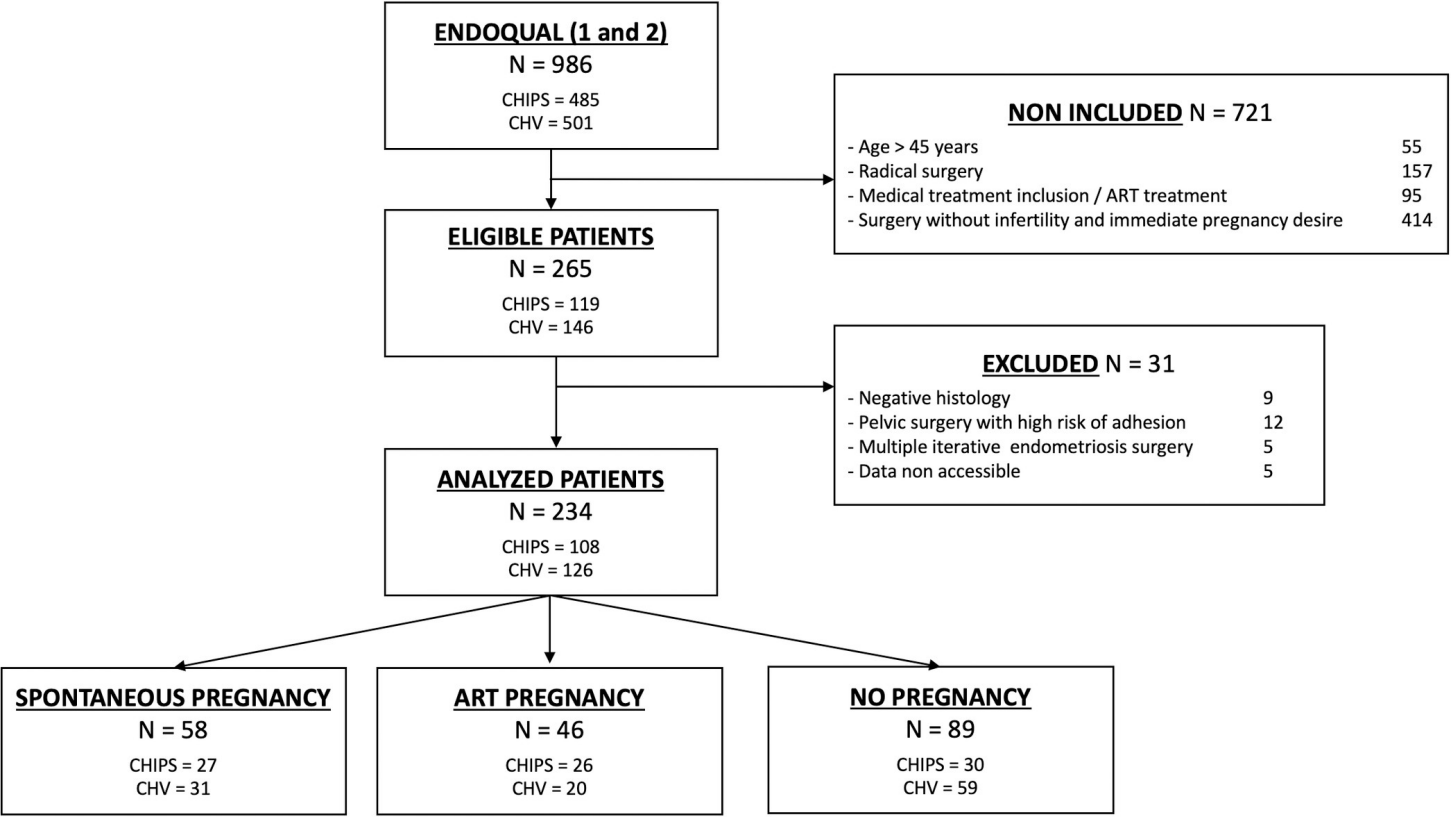
Flow chart. N, number of patients; CHIPS, Centre Hospitalier Intercommunal de Poissy St Germain; CHV, Centre Hospitalier de Versailles; EFI, Endometriosis Fertility Index.

During the study period, 986 patients were enrolled in the ENDOQUAL cohort. Among them 275 meet the inclusion criteria and du to exclusion criteria the final analysis included 234 patients with infertility or immediate desire to conceive after the surgery (Fig 1).

The patients’ clinical characteristics according to pregnancy status and conception mode are shown in Table 1. During the follow-up, 104/234 patients (44.4%) became pregnant, including 58 (55.8%) spontaneous conceptions and 46 (44.2%) obtained by ART. The mean follow-up among the patients who did not conceive was 29.7 months (± SD 24.7). For all pregnancies, mean conception time was 17 months (± SD 15.6). The time to conceive was lower for spontaneous conception compared to ART conception (14.3 months versus 20.4 months, p = 0.047, 95% CI [-12.1; -0.09]).

**Table 1:** Characteristics of study patients according to pregnancy status and conception mode.

	ALL PATIENTS	PREGNANT			NON PREGNANCY	p***
		Spontaneous	ART	p**		
	N = 234	N = 58	N = 46		N = 89	
	n (%)*	n (%)*	n (%)*		n (%)*	
**Mean Follow up** month (SD)	22.9 (21.2)	14.3 (12.7)	20.4 (18.2)		29.7 (24.7)	
**Mean Conception time** months (SD)	17 (15.6)	14.3 (12.7)	20.4 (18.2)	0.047		
**Age** Mean years (SD)	32 (4.7)	30.5 (4.8)	31.8 (4.3)	0.14	33 (4.7)	0.01
< 35 years	160 (68)	47 (81)	31 (68)	0.29	55 (62)	0.15
36–39 years	58 (25)	9 (16)	13 (28)		26 (29)	
> 40 years	16 (7)	2 (3)	2 (4)		8 (9)	
**Mean Infertility Time** months (SD)	32 (27)	23.2 (17.8)	30.5 (18.8)	0.12	38 (34)	0.05
< 3 years	170 (73)	46 (79)	30 (65)	0.17	63 (71)	0.27
> 3 years	64 (27)	12 (21)	16 (35)		26 (29)	
**Prior Pregnancy**						
Yes	77 (33)	16 (28)	10 (22)	0.65	32 (36)	0.21
No	157 (67)	42 (72)	36 (78)		57 (64)	
**AFS Total** Mean (SD)	43 (37.5)	32.3 (29.9)	53 (42)	< 0.01	51 (40)	< 0.01
< 71	179 (76)	49 (84)	32 (70)	0.11	62 (70)	0.10
> 71	55 (24)	9 (16)	14 (30)		27 (30)	
**ASRM**						
I	28 (12)	7 (12)	2 (4)	0.07	10 (11)	0.038
II	47 (20)	18 (31)	10 (22)		12 (13)	
III	64 (27)	17 (29)	10 (22)		22 (25)	
IV	95 (41)	16 (28)	24 (52)		45 (51)	
**AFS Endometriosis Score** Mean (SD)	14.2 (12.8)	11.7 (10.3)	16.5 (15.3)	0.07	15.2 (13.2)	0.16
**LF Score** Mean (SD)	5.3 (1.8)	5.8 (1.7)	5.1 (1.7)	0.03	5.1 (1.9)	0.04
[1–3]	38 (16)	5 (9)	9 (20)	0.10	18 (20)	0.16
[4–6]	139 (60)	34 (58)	29 (63)		53 (60)	
[7–8]	57 (24)	19 (33)	8 (17)		18 (20)	
**EFI** Mean (SD)	6 (2)	6.4 (1.6)	5.9 (1.9)	0.16	5.8 (2.1)	0.18
[0–2]	13 (5.5)	1 (2)	4 (9)	0.27	6 (7)	0.56
[3–4]	28 (12)	5 (9)	8 (17)		11 (12)	
[5–6]	93 (40)	25 (43)	14 (30)		39 (44)	
[7–8]	76 (32.5)	21 (36)	16 (35)		25 (28)	
[9–10]	24 (10)	6 (10)	4 (9)		8 (9)	
**Tabac**	40 (17)	7 (12)	4 (9)	0.37	17 (19)	< 0.01
**AMH** (ng/ml) Mean (SD)	4.4 (4.5)	5 (3.8)	4.8 (5.3)	0.85	3.4 (3.8)	0.27
**BMI** (kg/m2) Mean (SD)	23.3 (4.7)	23.2 (4)	23.7 (5.3)	0.63	23 (3.7)	0.67

* Results presented are numbers of patients (%) unless otherwise stated.

** Comparison of pregnant women by method of conception.

*** Comparison groups of pregnant women and non-pregnant women. Legend: ART, Assisted Reproductive Technology; SD, Standard Deviation; AFS, American Fertility Society; ASRM, American Society for Reproductive Medecine; LF, Least Function; EFI, Endometriosis Fertility Index; AMH, Anti-Müllerian hormone; BMI; Body mass index.

The mean a posteriori EFI calculated in patients from ENDOQUAL-1 was 6.1 (± SD 1.73) versus 5.6 (± SD 2.74) for the patients from ENDOQUAL-2, with no significant difference between the two results (p = 0.19 95% CI [-0.25; 1.22]). The mean total EFI was 5.9 (± SD 2.04).

### Cumulative incidence of all pregnancies with competing risks

**Fig 2:**
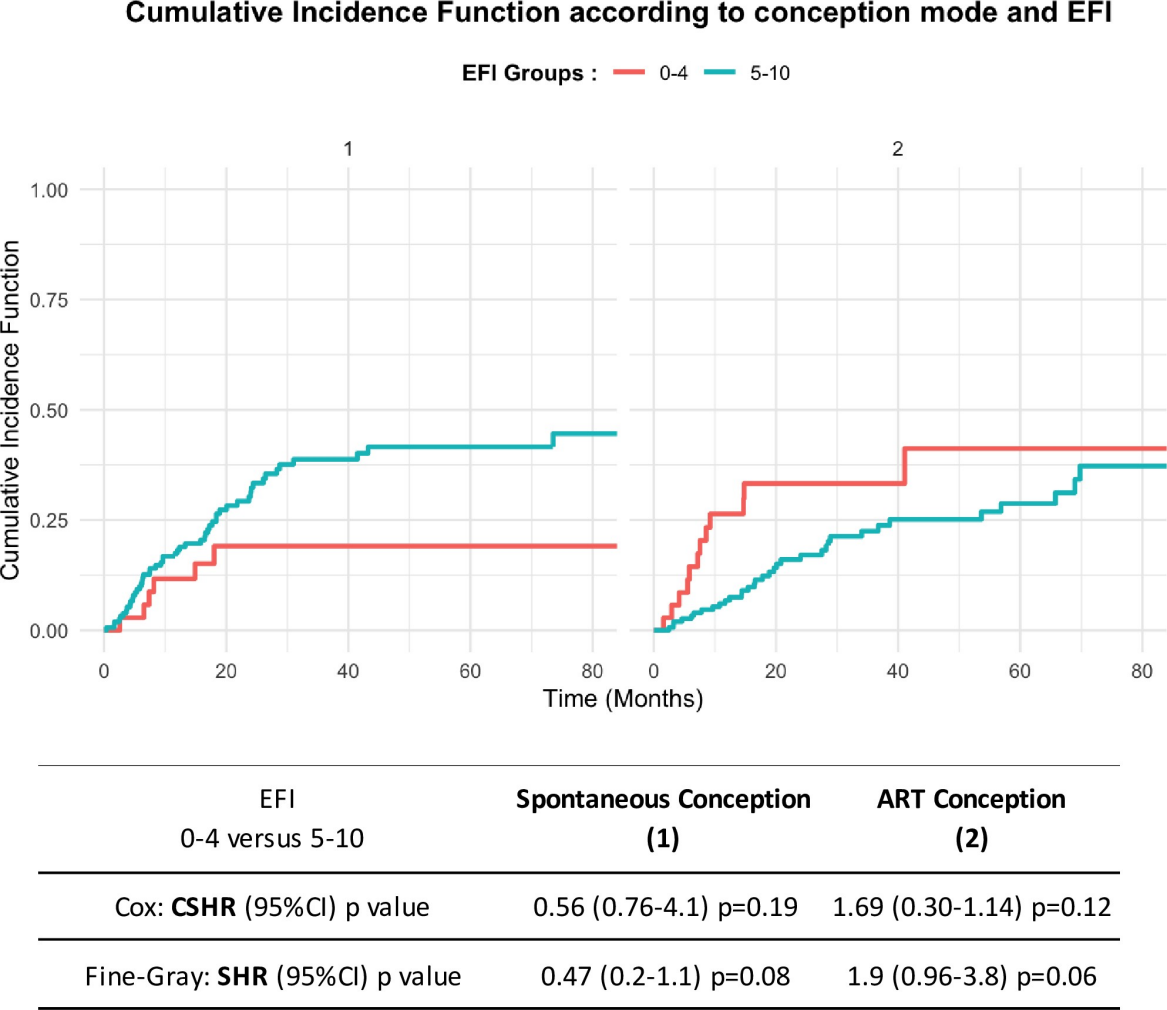
Cumulative incidence function according to conception mode and EFI with CSHR (Cause Specific Hazard Ratio) and SHR (Sub Distribution Hazard Ratio) analysis. ART, Assisted Reproductive Technology; CSHR, Cause Specific Hazard Ratio; SHR, Sub-distribution Hazard Ratio; IC, confidence interval; EFI, Endometriosis Fertility Index.

Cumulative incidence curves in the presence of competing risks revealed a higher rate of pregnancies by spontaneous conception in patients with an EFI of 5–10 (38.8%, 95% CI [25.3; 52.98] at 36 months versus 18.6%, 95% CI [0.42; 64.1] for an EFI of 0–4) and a higher rate of pregnancies by ART conception in patients with an EFI of 0–4 (33.3%, 95% CI [9.92; 65.11] of cumulative pregnancies at 36 months versus 22.5%, 95% CI [10.7; 41.17] for patients with an EFI of 5–10) (Fig 2).

Using the Fine and Gray model, the likelihood of a natural pregnancy tended to be lower in patients with an EFI of 0–4 compared with an EFI of 5–10 (SHR = 0.47, 95% CI [0.2; 1.1], p = 0.08).

Patients with an EFI of 0–4 had a higher success with an ART pregnancy than patients with a an EFI of 5–10. (SHR = 1.9, 95% CI [0.96; 3.8] p = 0.06). The results were consistent with those posted by the CSHR. The overall results were not significant.

### RMCT and fecundability

The difference between the two RMCT groups increased progressively over time with the main increase occurring 18–24 months postoperatively as shown in Table 2. At 36 months, patients with an EFI of 0–4 had lost 2.8 months (HR = 1.78, 95% CI = [0.76–4.16], p = 0.18) to achieve a pregnancy, without statistically significant difference.

**Table 2:** AUC and fecundability (%, 95%CI) according to EFI for spontaneous pregnancy.

Post Operative Interval	N	AUC			Fecundability	
		RMCT		Time Lost (months)	EFI score 0–4	EFI score 5–10
		EFI score 0–4	EFI score 5–10			
0–6 months	17	5,90	5,75	0,15	0.6 [-0.5; 1.7]	1.9 [1; 2.8]
6–12 months	15	5,32	5,08	0,24	2.4 [-0.3; 5.1]	1.8 [0.8; 2.8]
12–18 months	9	5,00	4,72	0,28	1.2 [-2.2; 3.6]	1.6 [0.5; 2.7]
18–24 months	7	4,48	4,19	0,29	0	2.1 [0.5; 3.7]
24–30 months	3	4,48	3,65	0,83	0	1.3 [-0.1; 2.7]
30–36 months	1	4,48	3,38	1,10	0	0.6 [-0.5; 1.7]
0–36 months	52	29,65	26,85	2,80	0.9 [0; 1.8]	1.8 [1.3; 2.7]

N, number of spontaneous pregnancies; AUC, Aera Under Curve; RMCT, Restricted Mean Conception Time; CI, Confidence Interval; EFI, Endometriosis Fertility Index.

Over 36 months postoperatively, the fecundability in patients with an EFI of 0–4 was 0.9% versus 1.8% for patients with an EFI of 5–10. The fecundability of patients with an EFI of 0–4 decreased from 12 months postoperatively. For patients with an EFI of 5–10, fecundability was stable until 24 months postoperatively, followed by a progressive decrease (Table 2). The probability of spontaneous conception for patients with an EFI of 0–4 was optimal between 6 and 12 months postoperatively.

## Discussion

As well as being a robust tool for predicting the pregnancy rate after surgery in women with endometriosis-related infertility, the EFI is also an important element to be considered in the therapeutic decision-making process. We observed more ART pregnancies in patients with an EFI of 0–4. Patients with a favorable EFI (≥5) had more natural pregnancies than patients with an unfavorable EFI (≤4). These results suggest that endometriosis patients with an EFI of 0–4 should be referred to an ART unit rapidly after surgery.

We found that fecundability was optimal up to 12 months postoperatively in women with an EFI ≤4, after which it decreased. For patients with an EFI ≥5, fecundability was stable up to 24 months postoperatively before decreasing. Fecundability indicates the time during which a surgeon should refer the patient to an ART unit. Therefore, according to our results, women with an EFI ≤4 should be referred within 12 months and those with an EFI ≥5 within 24 months. These results are confirmed by the AUC analysis. The loss of time for a spontaneous conception gradually increased postoperatively for women with an EFI of 0–4 versus those with an EFI of 5–10.

Our analysis was based on the ENDOQUAL study, an important bi-center prospective cohort of endometriosis patients allowing continual evaluation of health practices. As in other studies, regular reassessment by questionnaires and telephone interviews constitutes the usual reference techniques to monitor patients’ fertility [14, 15, 25].

One of the strengths of our study lies in the statistical model we used based on competing risk which is, to the best of our knowledge, innovative in this context. In their princeps article, Adamson and Pasta (2010) applied the gold standard for analyzing the effectiveness of infertility treatments, i.e., analysis by censored data and the survival curve method [22, 23]. However, the 801 patients analyzed in the article were operated on for infertility and not pain, which is not the case for all studies on the EFI [9, 26]. Recent advances in epidemiologic and biostatistical methods have made available multiple tools to describe differences in times to outcomes related to an exposure in this context [18, 21]. The competing risk model in our study gave similar results to the AUC analysis and fecundability for inter-group differences and intra-group evaluation, respectively.

One limitation of our study is the small number of patients in each group when we analyze time to conception. Nevertheless, despite imperfections from our population, our analysis allowed us to identify therapeutic profiles and determine an optimal care approach. Another limitation is that we lacked precise information about how long the patients actually attempted pregnancy taking into account the duration of postoperative hormonal treatment or post-operative pain, for example, which may have postponed attempts to conceive naturally. Furthermore, although all patients were allowed to attempt natural conception, we do not know precisely if women became pregnant naturally after ART failure or how long they were exposed to natural conception before being referred for ART. Nevertheless, the statistical analysis tool we used (i.e., SHR and Fine and Gray model) minimized the bias related to this issue.

The monthly conception rate (0% to 2.5%) in our population was lower than that of Marcoux et al. (1997) [27]. These authors demonstrated that fertility was improved in women who underwent resection for endometriosis lesions compared to those would did not (4.7% versus 2.4%; RR = 1.9, 95% = [1.2–3.1]). They focused their analysis on patients with mild to moderate stages of endometriosis (Stage I or II) [28]. Conversely, we included all endometriosis stages and 68% of our patients had stage III or IV. The low fecundability we observed in patients with an EFI of 0–4 can be explained by hormonal blockage observed postoperatively in patients with severe endometriosis.

Physiopathologically, our results can be explained by a beneficial effect of the surgery by restoring the pelvic anatomy. Patients with an EFI ≤4 may have residual endometriosis (incomplete surgery, a poor LF Score). Zhang et al. explained that visible endometriotic lesions are not sufficient to describe disease severity and that surgery cannot correct the associated molecular and immune phenomena [25]. However it might be understood there is an impact of endometriosis surgery on obstetrics outcomes, as Baggio et al. (2015) demonstrate [29].

With all these results, gynecologists may find it difficult to persuade women to opt for an expectant management, but clinical experience shows that patients prefer to attempt a natural conception. Surgery for endometriosis infertility was always to improve natural conception.

## General conclusion

Although the EFI is a valid tool to predict spontaneous pregnancy rates after surgery for endometriosis, using the EFI in routine clinical practice to triage patients remains a challenge. Our study suggests that the optimal time frame to manage postoperative infertility varies according to the EFI: patients with a favorable EFI (≥5) may be allowed to have 24 months of spontaneous attempts to conceive whereas patients with an unfavorable EFI (≤4) should be more rapidly referred for ART.

## Supporting information

S1 Data (XLSX)
